# The impact of work gamification on Chinese employee creativity: a moderated mediation model

**DOI:** 10.3389/fpsyg.2025.1494789

**Published:** 2025-07-02

**Authors:** Hui Liu, Jiachen Gao

**Affiliations:** International Business School, Shaanxi Normal University, Xi'an, China

**Keywords:** work gamification, intrinsic motivation, the level of acceptance of work gamification, creativity, flow theory

## Abstract

**Introduction:**

As economic and social development continues, the values and behavioral patterns within society are undergoing profound transformations, posing significant challenges to traditional enterprise management models. To enhance employee engagement and improve workplace efficiency, gamification design has increasingly gained attention in both theoretical discourse and practical enterprise management. This study aims to elucidate the mechanisms by which workplace gamification influences employee creativity, addressing urgent managerial challenges arising from the normalization of remote work and the increasing prevalence of Generation Z employees.

**Methods:**

Based on flow theory, this study analyzed survey data collected from 217 Chinese enterprise employees (84 males, 133 females; 83.87% aged 18–42) from January to February 2024. Intrinsic motivation was employed as the mediating variable, and employee acceptance of work gamification served as the moderating variable.

**Results:**

The findings reveal that work gamification significantly enhances employee creativity, with intrinsic motivation serving a pivotal mediating role. Furthermore, employee acceptance of work gamification moderates the relationship between gamification and intrinsic motivation; specifically, a higher level of acceptance strengthens the positive impact of gamification on intrinsic motivation. Additionally, acceptance level further moderates the mediating effect of intrinsic motivation on the relationship between work gamification and creativity. When employee acceptance is high, the indirect effect of gamification on creativity through intrinsic motivation is more pronounced.

**Discussion:**

The study offers substantial theoretical and practical contributions. Its findings enrich theoretical understanding regarding workplace gamification mechanisms (e.g., points, task challenges, virtual rewards) and boundary conditions, while also providing practical insights for enterprises aiming to effectively utilize gamification strategies to stimulate employee creativity.

## 1 Introduction

With the rapid development of the social economy, new-generation employees have become a vital component of enterprises. Raised in an era of economic globalization and rapid advancement in information technology, their values and behavioral patterns exhibit distinctive individual characteristics. Compared to previous generations, these employees place greater emphasis on deriving enjoyment from work and realizing personal value, moving beyond mere satisfaction of basic livelihood needs. This trend toward individualism has led to a growing misalignment between traditional work models and motivational management approaches. Consequently, effectively stimulating employee creativity has emerged as a critical challenge for enterprises navigating intense market competition.

Against this backdrop, work gamification has garnered increasing attention from both academia and practice. As an innovative work design strategy, it reconstructs workflows and behavioral patterns by integrating game design principles and interactive elements, making work more engaging and appealing (Florin et al., 2014). Existing research has preliminarily confirmed that work gamification can significantly enhance employee job satisfaction, engagement, intrinsic motivation, and innovative performance (Mollick and Rothbard, 2013; Robson et al., 2016; Behl et al., 2022; Friedrich et al., 2019; Cardador et al., 2017; Bitrián et al., 2023). However, most studies focus on the impact of gamification design on general workplace outcomes and psychological states, with relatively limited exploration of how gamification enhances employee creativity—particularly lacking systematic theoretical explanations and contextual analyses. Specifically, the precise internal mechanisms through which work gamification effectively stimulates creativity, the contextual conditions under which it operates, and how employee creativity is influenced by psychological states like intrinsic motivation remain unclear. Resolving these questions is crucial for deepening the understanding of gamification mechanisms, defining effective applicability conditions, and providing guidance for management practices, necessitating further research.

Grounded in flow theory, this study examines the mechanism by which work gamification influences employee creativity. Flow theory emphasizes that individuals achieve an optimal experiential state during activities, effectively promoting intrinsic motivation and thereby enhancing creativity (Csikszentmihalyi, 1991; Hamari and Koivisto, 2014). Work gamification fosters an ideal environment for flow experiences—through clear goals, immediate feedback, and balanced skill-challenge alignment—stimulating intrinsic motivation and subsequently elevating creativity levels. Furthermore, this study introduces the level of acceptance of work gamification as a moderating variable, positing that employees' level of acceptance significantly influences the effectiveness of flow experiences in stimulating intrinsic motivation (Mitchell et al., 2020). Employees with a higher level of acceptance are more likely to actively engage with gamified designs, yielding greater creativity enhancement; conversely, effects are constrained when the level of acceptance is low. Thus, investigating how the level of acceptance of work gamification moderates the relationship between gamification and creativity constitutes a key focus of this research.

In summary, this study explores the impact mechanism of work gamification on employee creativity based on flow theory, revealing the mediating role of intrinsic motivation and the moderating effect of the level of acceptance of work gamification. It provides theoretical support and practical insights for understanding the effective mechanisms and real-world application of work gamification. The primary contributions are 3-fold: First, it constructs a theoretical model of how work gamification influences employee creativity, expanding the application scope of gamification research; Second, it employs flow theory as the foundational framework, deepening the theoretical understanding of gamification incentive mechanisms; Third, through empirical analysis, it validates the mediating effect of intrinsic motivation and the moderating role of the level of acceptance, offering scientific evidence and practical guidance for enterprises implementing work gamification strategies.

## 2 Theoretical framework and hypotheses development

### 2.1 Hypotheses formulation

#### 2.1.1 Work gamification and creativity

The term “gamification” was first proposed in 2003 by British game developer Nick Pelling. Deterding et al. (2011) defined gamification from a systems design perspective as “the use of game design elements in non-game contexts to create distinctive gamified experiences that drive behavioral change.” With the rapid advancement of information technology, the application scope of gamification has expanded extensively into fields such as business, education, and healthcare. In education, Dichev and Dicheva (2017) conducted a critical literature review on educational gamification, noting that practical implementations of mechanisms like Points, Badges, and Leaderboards significantly predated academic understanding of their underlying mechanisms. However, empirical research remains insufficient and exhibits pronounced disciplinary bias—studies are predominantly concentrated in computer science, with minimal representation in humanities and social sciences. Sailer and Homner (2020) performed a meta-analysis of 38 studies and found that gamification exerts statistically significant, albeit modest, effects on three categories of learning outcomes: cognitive, motivational, and behavioral. In medical and health contexts, Johnson et al. (2016) synthesized prior research to indicate that gamification positively influences health behaviors—particularly physical activity—though its impact on cognitive outcomes remains unclear. Sardi et al.'s (2017) review emphasized that gamification finds its most extensive applications in chronic disease management (e.g., diabetes rehabilitation) and physical activity motivation, with reward systems and real-time feedback being the most frequently employed game mechanisms. In marketing, Xi and Hamari (2019) validated, based on self-determination theory, that gamification enhances brand engagement by fulfilling users' needs for autonomy, competence, and relatedness. Synthesizing the aforementioned findings and scholars' research on gamification and creativity in work contexts, scholarly research posits that gamification in the workplace can enhance employee performance and invigorate their intrinsic motivation. Academic discourse defines work gamification as the deliberate integration of game design elements into work processes, transforming the work experience into one that resembles gaming (Florin et al., 2014). The implementation of gamification enhances employee engagement, facilitates the setting of clear objectives and the provision of timely feedback, thereby fostering their innovative contributions within practical work environments (Patrício et al., 2018). Engagement with game elements enhances employee creativity and expands the application of their innovative ideas. Furthermore, gamification enhances internal communication, encouraging employees to think and act innovatively, thereby driving innovation in both products and processes.

Work gamification aims to create a flow experience for employees. Jobs that incorporate game elements such as diversity, adaptive challenges, clear objectives, and immediate feedback become more engaging for employees across different skill levels. Employing work gamification strategies, companies can create innovative workplace environments conducive to skill development, unleashing employees' creative potential. This kind of job design not only optimizes the intrinsic motivation of employees but also brings innovative momentum to the organization.

Based on the above analysis, this paper hypothesizes that:

Hypothesis 1: Work gamification has a positive impact on employee creativity.

#### 2.1.2 Work gamification and intrinsic motivation

Work gamification, as a motivational strategy, can catalyze significant behavioral changes within individuals. It carefully combines extrinsic incentives with intrinsic motivations to drive employee actions, thereby achieving desired outcomes and fulfilling employees' intrinsic needs. Research indicates that by incorporating game-like elements into tasks, gamification enhances the enjoyment derived from work-related activities (Cardador et al., 2017). The achievement of a flow state depends on several key conditions: clear objectives, immediate and transparent feedback, and a balance between task difficulty and individual skill levels (LoVoll and Vitters, 2014; Palomki et al., 2021). These conditions are closely related to the stimulation of intrinsic motivation.

Firstly, gamification mitigates the constraints typically encountered in real-world scenarios, thereby bolstering individual autonomy by offering a diverse array of choices. This power of autonomous choice not only reflects the player's personal will but is also a significant factor in stimulating their intrinsic motivation (Smith and Popa, 2015; Gulzar et al., 2021; Hagtvedt et al., 2019; Silvia et al., 2008). Secondly, the immediate feedback provided by game systems has a notable impact on players' sense of competence. This feedback not only reinforces players' behavior but also strengthens their expectation of success and sense of control, thereby satisfying their need for recognition of their abilities and self-efficacy (Amabile et al., 2005; Han et al., 2019). Lastly, social interactions within games are crucial for meeting individuals' needs for belonging and social connection. By cooperating and competing with other players, individuals can build social bonds. These interactions not only enliven the player's gaming experience but also facilitate the fulfillment of social needs, supplying players with social support and a sense of communal identity.

In conclusion, gamification adeptly addresses individuals' psychological needs for autonomy, mastery, and social connection, thereby enriching their intrinsic motivation and overall gaming experience through the enhancement of choice autonomy, provision of immediate feedback, and cultivation of social interaction. These factors work together on the player's intrinsic motivation and gaming experience. Integrating gamification into the workplace can augment employee engagement with their tasks, thus elevating their intrinsic motivation.

Based on the above analysis, this paper hypothesizes that:

Hypothesis 2: Work gamification has a positive effect on employees' intrinsic motivation.

#### 2.1.3 Intrinsic motivation and creativity

Intrinsic motivation refers to the behavior of an individual to engage in an activity for its inherent interest and sense of satisfaction, without the presence of external rewards (Mitchell et al., 2020). As indicated by flow theory, the experience of flow is a reflection of intrinsic motivation, wherein individuals persist in their participation due to the activity's inherent appeal during a flow state. This engagement is fundamentally rooted in intrinsic motivation (Jiang et al., 2021). Individuals in a state of flow exhibit a stronger intrinsic motivation compared to when they are not in a flow state (Keller and Landhaeusser, 2011). Flow theory emphasizes the alignment of task difficulty with individual capability and the provision of immediate feedback as essential catalysts for both stimulating and sustaining intrinsic motivation. Moreover, flow experiences are typically associated with intrinsic motivation and curiosity (Csikszentmihalyi, 1991). Intrinsic motivation impels employees to venture into the unknown and exhibit a robust interest in novel knowledge, skills, and concepts. This desire for novelty is the starting point for creative thinking. Research indicates that curiosity and creativity are complementary and mutually reinforcing (Schutte and Malouff, 2020; Jie and Wenyuan, 2023; Kang and Zheng, 2023). Additionally, research on intrinsic motivation points out that fulfilling an individual's basic psychological needs—such as autonomy, competence, and relatedness—is crucial for stimulating intrinsic motivation (Deci and Ryan, 2013). And these needs are closely connected to the characteristics of flow experiences (Meng, 2016). When individuals recognize their capacity to tackle challenges and maintain control over their endeavors, they are more inclined to pursue challenging activities conducive to learning and personal development. This confidence helps individuals persist in the long term and creatively solve problems (Amabile et al., 1994; Marylène and Deci, 2005). Thus, satisfying these psychological needs enhances an individual's intrinsic motivation, which in turn fosters the development of creative thinking and innovative behavior.

Based on the above analysis, this paper hypothesizes that:

Hypothesis 3: Employees' intrinsic motivation has a positive impact on their creativity.

#### 2.1.4 The mediating role of intrinsic motivation

Intrinsic motivation is the driving force that originates from an individual's inherent interest and the enjoyment they derive from an activity. Human behavior is fundamentally propelled by motivation, which is rooted in underlying needs. Deci and Ryan (2013) proposed self-determination theory, defining intrinsic motivation as “acting for inherent satisfaction” and establishing three fundamental psychological needs: autonomy, competence, and relatedness. In workplace contexts, employees' intrinsic motivation often stems from satisfaction derived from gamified designs. Ryan and Deci (2000) systematically integrated the self-determination framework to advance the “golden triangle” of intrinsic motivation—autonomy, competence, and relatedness needs. Gursesli et al. (2024) developed and validated a Psychological Motives for Playing Video Games (PMPVGs) scale applicable across game genres, addressing limitations of prior motivation measures tied to specific game types. The final validated instrument comprises 12 dimensions, all exclusively focused on intrinsic psychological needs. This demonstrates that gamification can influence players' motivations, thereby establishing connections with motivation-related outcomes. In the workplace, employees' intrinsic motivation often originates from the satisfaction associated with gamified design elements. Banfield and Wilkerson (2014) observed that incorporating gamified elements into education can significantly enhance students' intrinsic motivation. Bakker and Demerouti (2007) found that engaging in challenging activities can elicit a strong intrinsic motivational response, prompting employees to exhibit increased intrinsic motivation. Chen and Gao (2021) conducted empirical research showing that work gamification has a substantial positive effect on employees' sense of workplace spirituality. In 1975, cognitive psychologist Csikszentmihalyi formally proposed flow theory, describing a state in which individuals become fully immersed in an activity, experiencing a transcendence of temporal and spatial awareness. The enjoyment and satisfaction derived from such activities significantly exceed expectations, resulting in an optimal experience termed “flow”—a profound sense of pleasure and fulfillment arising from deep immersion (Csikszentmihalyi, 1991). Within the field of education, Kiili introduced the “Experiential Gaming Model,” integrating flow theory into educational game design. This framework emphasizes maintaining challenge-skill balance through dynamic difficulty adjustment (Kiili, 2005). Building upon flow theory, Shanshan (2025) applied these principles to optimize practical teaching components in tourism higher education, aiming to enhance student engagement and skill mastery. Flow theory asserts that individuals immersed in a state of flow are primarily motivated by passion and curiosity, transcending the pursuit of external rewards. Individuals experiencing flow exhibit a high degree of autonomy, competence, and connectedness, all of which are key factors of intrinsic motivation. When social contexts and events adeptly fulfill these three fundamental psychological needs, they are more apt to evoke a heightened level of intrinsic motivation. Gamified characteristics substantially address these needs, allowing employees the autonomy to choose participation, timing, and degree of engagement; the gamification process features clear objectives and prompt feedback. Recognizing the value and rewards of their work, employees are inclined to work with greater diligence and devotion, showcasing enhanced creativity (Fuller and Hester, 2010; Yidong and Xinxin, 2013). Novak and Hoffman delineate the concept of flow into three distinct phases: flow antecedents, flow experience, and flow outcomes. The pursuit of achieving a flow state, particularly meeting the antecedent conditions, remains a pivotal area of scholarly exploration. Flow antecedents encompass clear goals, timely feedback, and a match between skill and challenge. In the phase of flow antecedents, an individual's intense interest in the task, coupled with the formulation of precise and clear objectives, is instrumental in reaching the flow state. The continuity of the flow experience is essential and relies on the provisioning of immediate feedback. Work gamification sustains the flow experience by ensuring immediate feedback and setting clear goals. In a state of flow, individuals are deeply immersed in the activity, impervious to external distractions and largely oblivious to the passage of time. A flow experience arises from a congruence between an individual's skill level and the task's difficulty. Furthermore, sustaining such an experience necessitates the continuous enhancement of one's skills to tackle escalating challenges (Novak et al., 2000). Moreover, gamified networks effectively interconnect employees' social networks, fostering a collaborative and competitive work environment. During this process, employees experience not only autonomy and competence in their tasks but also fulfill their social needs. Intrinsic motivation is aroused when employees' core needs are met, and they perceive a sense of acceptance and respect, which positively affects their creative performance. Therefore, intrinsic motivation acts as a key mediator between work gamification and employee creativity, indicating that work gamification enhances creativity through its influence on intrinsic motivation.

Based on the above analysis, this paper hypothesizes that:

Hypothesis 4: Intrinsic motivation mediates the relationship between work gamification and employee creativity.

#### 2.1.5 The modulating influence of the level of acceptance of work gamification

The level of acceptance of work gamification indicates the extent to which employees engage with and support the incorporation of gaming elements and principles into their work routines and tasks. This acceptance reflects an individual's willingness to acknowledge and endorse the introduced elements. Empirical studies have consistently demonstrated that work gamification positively affects various aspects of employees' work, including their psychological states, performance, and behavior. Glynn (1994) noted that incorporating gamified design into corporate practices can significantly increase employee interest and strengthen goal orientation. The acceptance of work gamification by individuals is crucial, as it influences the likelihood of achieving a flow state and indirectly stimulates intrinsic motivation and creativity. Key antecedents of flow include clear goals, timely feedback, and a balance between skill and challenge. Individuals with an open mindset toward work gamification are likely to find challenges that match their skills. They experience a sense of achievement through timely feedback. Such individuals also become deeply engaged in their work, directed by clear goals (Chen et al., 2000). This high level of engagement is central to the flow experience, allowing individuals to fully immerse themselves in tasks, disregard external distractions, and enjoy the work process itself. In a state of flow (Novak et al., 2003), individuals can not only enhance work efficiency and quality but also derive pleasure and satisfaction from task completion, which are significant sources of intrinsic motivation. As intrinsic motivation strengthens, individuals become more inclined to explore new methods and innovative ideas, thereby stimulating and enhancing their creativity. Moreover, gamified work designs, by increasing fun and participation, can spark employees' curiosity and desire to explore, further promoting the occurrence of flow experiences (Hung et al., 2015; Inal and Cagiltay, 2007; Wangsupa and Chensupa, 2010). Therefore, developing and implementing work gamification strategies requires careful consideration of individual differences and the prerequisites for flow experiences to optimize the potential for bolstering employees' intrinsic motivation and creativity. Maurer, a management professor at The Wharton School, collaborated with colleagues to design an engaging online sales game named “Dunk.” The game simulated a rapidly growing e-commerce company managing a sales force of hundreds, with large screens and leaderboards dynamically displaying sales efforts, symbolized by “dribbling,” “layup,” and “scoring” for lead acquisition, telephone sales, and deal closures. Maurer assessed participants' “identification” by measuring their acceptance of the game through surveys that included questions about attention, understanding of rules, and perceptions of fairness. His findings showed that strong identifiers experienced heightened positive emotions and improved attitudes toward the company. In contrast, a lack of identification led to negative emotions toward work and a decline in performance for some participants. Maurer considers the experiment significant for two reasons: it demonstrated gamification's substantial impact through a large-scale controlled study and also revealed that a lack of identification could diminish performance and attitudes. For employees who do not embrace gamification, the inherent enjoyment becomes contrived. Gamification strategies can enhance online training by increasing employee engagement; high acceptance of gamified components improves perceptions of usefulness and usability, further boosting interest and enjoyment (Bitrián et al., 2023). Employee acceptance significantly influences the capacity of gamification to foster positive psychological motivation; acceptance is essential for improving work motivation and stimulating innovative behavior.

Based on the above analysis, this paper hypothesizes that:

Hypothesis 5: The level of acceptance of work gamification moderates the relationship between the degree of work gamification and intrinsic motivation, such that employees with a higher level of acceptance of work gamification exhibit stronger intrinsic motivation as the degree of work gamification increases, compared to those with a lower level of acceptance.

#### 2.1.6 Moderated mediation framework

This study presents a moderated mediation framework, positing that the acceptance of work gamification moderates the mediating effect of intrinsic motivation on the relationship between work gamification and employee creativity. Specifically, employees who exhibit a greater acceptance of work gamification are more likely to experience increased intrinsic motivation, which in turn leads to enhanced levels of creativity compared to those with lower acceptance. Employees with a high acceptance of work gamification often have a more open and affirmative attitude toward gamified designs. This positive mental state facilitates their full immersion in gamified work contexts, leading to greater enjoyment and satisfaction. Zikos et al. (2019) found that the higher the employees' acceptance of gamification and knowledge-sharing platforms, the more actively they engage in knowledge exchange and sharing. According to flow theory, individuals are more likely to enter a state of flow when their skill level matches the challenges they face (Tian and Ou, 2023). In this state, individuals can fully leverage their potential and apply their knowledge and skills to the fullest. For employees who highly accept work gamification, the clear goals and timely feedback provided by gamified designs meet the conditions necessary for a flow experience. Consequently, they are more likely to achieve a state of selfless dedication at work, stimulating a stronger intrinsic motivation. The enhancement of intrinsic motivation further promotes the expression of employee creativity. When employees possess a strong intrinsic motivation at work, they tend to exhibit increased curiosity, willingness to innovate, and sustained engagement, all of which are vital sources of creativity. High-acceptance employees in a state of flow can focus intensely and immerse themselves completely in work tasks. This focus allows them to delve deeply into problems, break through mental stereotypes, seek solutions from multiple perspectives, and ultimately produce creative outcomes.

In conclusion, the level of employee acceptance of work gamification significantly influences its practical application by determining the potential for entering a flow state, which in turn moderates the positive impact of work gamification on intrinsic motivation. The enhancement of intrinsic motivation further promotes the improvement of employee creativity levels. This logical sequence underscores the significance of individual differences among employees in the implementation of work gamification.

Hypothesis 6: The level of employee acceptance of work gamification positively moderates the mediating role of intrinsic motivation in the relationship between work gamification and employee creativity. That is, the higher the level of acceptance of work gamification by employees, the stronger the indirect positive effect of work gamification on creativity through intrinsic motivation.

**Figure 1:**
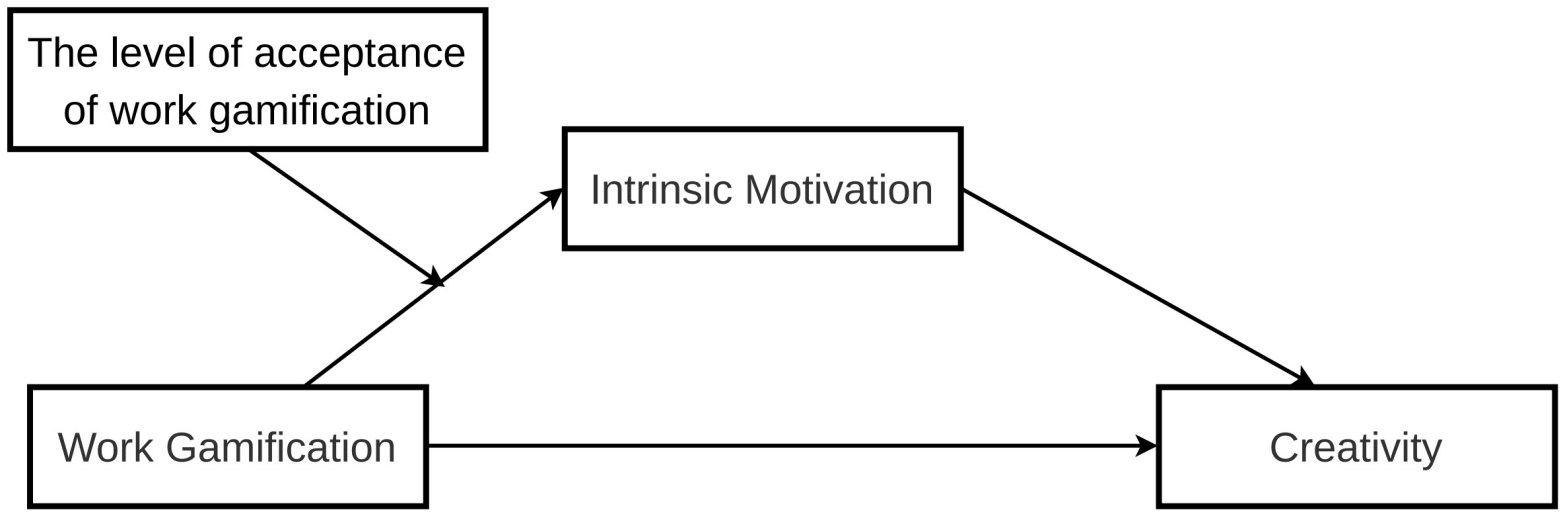
Theoretical model.

The theoretical model of this study is shown in Figure 1 below:

## 3 Methods

### 3.1 Data collection and research samples

This study, conducted between January and February 2024, utilized the Credamo research platform and targeted a sample of full-time employees from various industries in China. An online questionnaire was employed for data collection. To ensure data quality, researchers meticulously screened the responses, discarding those that exhibited obvious patterning or were completed unusually quickly. This rigorous process resulted in a total of 217 valid questionnaires for analysis.

To verify sample adequacy, this study employed the following two criteria: (1) Statistical power—Following Cohen's (1992) guidelines for multiple regression with seven predictors, an expected medium effect size (*f*^2^ = 0.15), significance level α = 0.05, and statistical power (1 – β) ≥ 0.80, the minimum required sample size is 103. Our sample size of 217 substantially exceeds this threshold. (2) Structural equation modeling heuristics—Bentler and Chou (1987) recommend a sample size no smaller than 5 times the number of free parameters. Our four-factor model contained ~35 free parameters, corresponding to a minimum requirement of ~175. Additionally, Comrey and Lee (1992) classify *N* ≥ 200 as “good,” collectively confirming that 217 samples are adequate and valid. Furthermore, dual-layer screening via IP addresses and response times during questionnaire collection further ensured sample quality.

Demographic variables of the 217 valid samples were analyzed using SPSS 26.0 software, the sample comprised 84 male and 133 female employees, with those aged 42 or younger accounting for 83.9% of the dataset. Bachelor's degree holders represented 70.9% of participants. The study covered diverse company types—including foreign-funded enterprises, state-owned enterprises (SOEs), private firms, and joint ventures—as well as organizations of varying scales from small to large enterprises, ensuring reasonable sample representativeness. While this survey did not differentiate between specific industries or job positions, future research could refine this aspect. With the results presented in Table 1.

**Table 1:** Demographic variable analysis.

**Characteristics**	**Feature distribution**	**Frequency**	**Percentage**	**Cumulative percentage**
Gender	Male	84	38.7	38.7
	Female	133	61.3	100.0
Age	18–28	98	45.2	45.2
	29–42	84	38.7	83.9
	43–58	12	5.5	89.4
	Over 59 years old	23	10.6	100.0
Education	Associate degree or below	35	16.1	16.1
	Bachelor's degree	119	54.8	70.9
	Master's degree	51	23.6	94.5
	Doctoral	12	5.5	100.0
Company size	<50 people	46	21.2	21.2
	50–200	62	28.6	49.8
	200–500	45	20.7	70.5
	500–1,000	28	12.9	83.4
	More than 1000	36	16.6	100.0
Company nature	Foreign enterprises	34	15.7	15.7
	State-owned enterprises	43	19.8	35.5
	Private enterprises	84	38.7	74.2
	Joint Venture	56	25.8	100.0

### 3.2 Measurements

This study employed established English scales developed by domestic and international scholars. Although Chinese versions exist for some scales, these translations primarily relied on student samples, and certain items were incongruent with the corporate context central to this research. To ensure semantic equivalence and cultural appropriateness, all English scales underwent localized adaptation following Brislin's (1986) translation-back translation protocol. The specific procedure was as follows: Forward Translation: One bilingual management scholar holding a Ph.D. translated the original scales into simplified Chinese; Expert Review: Two bilingual researchers with expertise in work gamification critically evaluated each item and proposed revisions; Back Translation: An independent bilingual management scholar, uninvolved in the initial translation, retranslated the revised Chinese version into English; Consistency Evaluation: The research team collectively compared the back-translated version with the original, resolving semantic discrepancies to finalize the scales. The adapted scales demonstrated good reliability (Cronbach's α > 0.70) in a pilot test, confirming measurement instrument robustness. All items were measured using a 5-point Likert scale (1 = “strongly disagree”, 5 = “strongly agree”).

#### 3.2.1 Work gamification

The study integrates existing content on the design of work gamification and adopts the scale developed by Koivisto and Hamari (2019). This scale comprises items such as “My work clearly informs me of the goals to be achieved and the current progress of my work,” “There are leaderboards in my work that allow me to compare and compete with others based on recent performance,” and 12 additional items, measuring the gamification of employees' work in enterprises. The Cronbach's α coefficient for this scale in this study is 0.772.

#### 3.2.2 Creativity

There are numerous scales for measuring creativity, but this paper has chosen the creativity scale developed by Danis and Dollinger (1998), which includes items such as “When I encounter difficulties, I can always think of alternative ways to solve them,” “I always need a variety of stimuli to generate new ideas,” and seven other items. In this study, the Cronbach's α coefficient for this scale is 0.624.

#### 3.2.3 Intrinsic motivation

The measurement of intrinsic motivation is derived from the scale developed by Tierney et al. (1999), which includes items such as “I enjoy finding ways to solve challenging problems,” “I like to come up with new ideas for the work I do,” and five other items. In this study, the Cronbach's α coefficient for this scale is 0.860.

#### 3.2.4 The level of acceptance of work gamification

The measurement of acceptance of gamification elements utilizes a scale developed by scholar Liu (2017) in his work “Modeling and Practical Application Verification of Gamified Learning Activities,” which includes items such as “When playing games, I pay attention to the completion of levels/segments,” “When playing games, I pay attention to the conditions for clearing the game,” and 12 other items. In this study, the Cronbach's α coefficient for this scale is 0.840.

#### 3.2.5 Control variables

Basic demographic characteristic variables such as gender, age, education level, company size, and company nature are treated as control variables.

**Figure 2:**
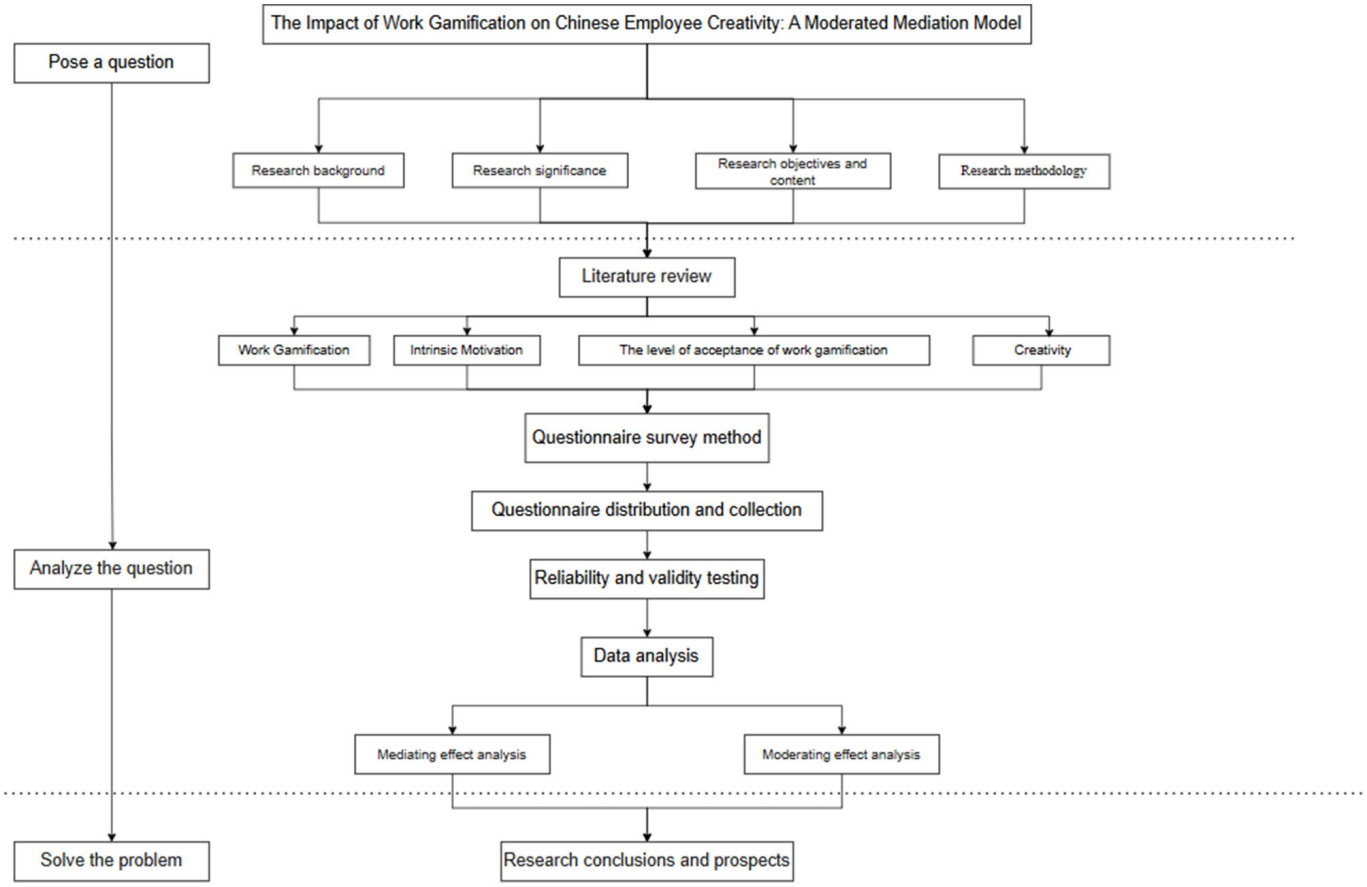
Technical roadmap.

Furthermore, to better present the research content, subjects, methodology, theoretical framework, etc., a technical roadmap is illustrated in Figure 2. This enhances the visual representation of the study.

## 4 Results

### 4.1 Descriptive statistical analysis

The study employs a 5-point Likert scale, ranging from a minimum of 1 to a maximum of 5, for respondent evaluation of all research variables on the survey questionnaire. Hypothesis testing is predicated on establishing data normality through skewness and kurtosis coefficients before analyzing the collected data. Descriptive statistical analysis of the research variables is presented in Table 2. Table 2 indicates that all items' skewness and kurtosis fall within acceptable limits (skewness absolute value < 3, kurtosis absolute value < 10), confirming normal data distribution and justifying further analysis.

**Table 2:** Descriptive statistics for study variables.

**Item**	**Mean**	**Std**.	**Skewness**	**Skewness S.E**.	**Kurtosis**	**Kurtosis S.E**.
CZL1	3.871	0.9289	−1.139	0.165	1.704	0.329
CZL2	3.926	0.8841	−0.626	0.165	−0.009	0.329
CZL3	3.922	0.9663	−0.899	0.165	0.743	0.329
CZL4	3.760	0.9849	−0.530	0.165	−0.203	0.329
CZL5	3.876	0.9468	−0.838	0.165	0.767	0.329
CZL6	3.797	0.9552	−0.709	0.165	0.330	0.329
CZL7	3.751	1.0242	−0.659	0.165	−0.065	0.329
IM1	4.046	0.8648	−0.653	0.165	−0.007	0.329
IM2	4.060	0.8450	−0.811	0.165	0.513	0.329
IM3	4.152	0.7453	−0.593	0.165	0.046	0.329
IM4	4.018	0.8922	−0.786	0.165	0.387	0.329
IM5	4.037	0.8436	−0.631	0.165	0.311	0.329
YXH1	4.014	0.9452	−1.024	0.165	1.175	0.329
YXH2	3.719	0.9667	−0.496	0.165	−0.284	0.329
YXH3	3.912	0.9213	−0.936	0.165	0.854	0.329
YXH4	3.825	1.0169	−0.762	0.165	0.148	0.329
YXH5	4.014	0.9976	−1.073	0.165	0.877	0.329
YXH6	3.940	0.9724	−1.130	0.165	1.385	0.329
YXH7	3.774	1.0362	−0.569	0.165	−0.412	0.329
YXH8	4.078	0.8810	−1.015	0.165	1.171	0.329
YXH9	3.959	1.0243	−1.012	0.165	0.564	0.329
YXH10	3.756	1.0320	−0.720	0.165	−0.044	0.329
YXH11	3.889	0.9213	−0.925	0.165	0.975	0.329
YXH12	4.028	0.9225	−0.948	0.165	1.037	0.329
JS1	4.147	0.8800	−1.033	0.165	1.140	0.329
JS2	4.055	0.9845	−0.993	0.165	0.784	0.329
JS3	3.903	0.9304	−1.059	0.165	1.455	0.329
JS4	3.945	0.9845	−0.858	0.165	0.421	0.329
JS5	4.055	0.9797	−1.185	0.165	1.385	0.329
JS6	4.005	0.9789	−0.995	0.165	0.849	0.329
JS7	3.986	0.9788	−0.779	0.165	0.038	0.329
JS8	4.120	0.9547	−1.112	0.165	0.981	0.329
JS9	3.991	0.9718	−0.867	0.165	0.254	0.329
JS10	4.078	1.0401	−1.055	0.165	0.545	0.329
JS11	3.995	0.9001	−0.914	0.165	0.819	0.329
JS12	4.032	0.9878	−1.083	0.165	1.090	0.329

### 4.2 Test on reliability and validity

Table 3 presents Cronbach's α coefficients for the variables of work gamification, creativity, intrinsic motivation, and acceptance of work gamification at 0.772, 0.624, 0.860, and 0.840, respectively. With all values exceeding 0.6, the scales demonstrate satisfactory overall reliability. Table 4 illustrates that the four-factor model outperforms alternative models, with the optimal fit indices (χ2 = 932.887, df = 588, RMSEA = 0.052, CFI = 0.861, TLI = 0.852; SRMR = 0.055), surpassing the fit of three-factor, two-factor, and one-factor models. This affirms the discriminant validity of the study's variables. Although the Cronbach's α for the creativity scale was marginally below 0.7, confirmatory factor analysis demonstrated good overall fit for the four-factor measurement model (see Table 4). This indicates acceptable construct validity across all scales, including the creativity measure, thus its results remained suitable for analysis in this study.

**Table 3:** Assessment of reliability and validity.

**Variable**	**Questions**	**Cronbach's α**
Work gamification	12	0.772
Creativity	7	0.624
Intrinsic motivation	5	0.860
The level of acceptance of work gamification	12	0.840

**Table 4:** Model fit comparison.

**Model**	**χ^2^**	**df**	***Δχ*^2^ (Δdf)**	**RMSEA**	**CFI**	**TLI**	**SRMR**
Four factor model	932.887	588	16.993 (3)^***^	0.052	0.861	0.852	0.055
Three factor model	949.888	591	26.808 (2)^***^	0.053	0.856	0.846	0.055
Two factor model	976.696	593	3.71 (1)^***^	0.055	0.846	0.836	0.056
Single factor model	980.406	594		0.055	0.845	0.835	0.056

*N* = 217, same below.

*** Indicates *p* < 0.001. In the four-factor model, each variable is independently loaded onto its respective factor. The three-factor model integrates work gamification with intrinsic motivation. The two-factor model combines work gamification, the level of acceptance of work gamification, and intrinsic motivation. The one-factor model merges all variables into a single construct.

### 4.3 Common method bias

To bolster the study's data quality and reliability, stringent controls were applied, including a clear research purpose in the questionnaire header, assurances of anonymity and confidentiality, and a commitment to academic use only, ensuring employees' personal information remains undisclosed. To mitigate social desirability bias, the questionnaire explicitly stated, “There are no right or wrong answers; respondents are encouraged to answer truthfully.” This approach aimed to reduce pressure stemming from social expectations or face-saving concerns, thereby mitigating the tendency to provide embellished responses. Additionally, adhering to academic standards, the data underwent an initial Harman single-factor test. The test results showed no single dominant factor, with the primary component explaining 31.332% of the variance—below the threshold of 40.00%. This indicates that common method bias exerted no significant influence on the study's findings.

### 4.4 Correlation analysis

Table 5 presents the correlation coefficients among the study's variables. The analysis reveals a strong positive correlation between work gamification and employee creativity, with a coefficient of 0.771 (*p* < 0.01); and between work gamification and intrinsic motivation, a coefficient of 0.636 (*p* < 0.01), also denoting a significant positive correlation, further, a correlation of 0.661 (*p* < 0.01) exists between intrinsic motivation and employee creativity, indicating a significant positive link. These findings offer preliminary support for the study's hypotheses.

**Table 5:** Correlations among variables.

**Variable**	**1**	**2**	**3**	**4**	**5**	**6**	**7**	**8**	**9**
1 Gender	1								
2 Age	−0.015	1							
3 Education	−0.028	−0.067	1						
4 Company size	0.021	0.153	0.171	1					
5 Company nature	0.051	−0.085	−0.087	−0.146	1				
6 Creativity	0.111	−0.185	0.065	0.015	−0.115	1			
7 Intrinsic motivation	0.094	−0.006	0.042	0.051	−0.1	0.661^**^	1		
8 Work gamification	0.088586	−0.295^**^	0.101322	0.05603	−0.11141	0.771^**^	0.636^**^	1	
9 The level of acceptance of work gamification	0.110529	−0.317^**^	0.058028	−0.00149	−0.05668	0.724^**^	0.570^**^	0.778^**^	1

^*^indicates *p* < 0.05,

** *p* indicates *p* < 0.01,

^***^p indicates *p* < 0.001, and this convention applies throughout this paper.

### 4.5 Hypotheses testing

#### 4.5.1 Main effects and mediation effect testing

The study initially performed a regression analysis, detailing the findings in Table 6. Controlling for gender, education, age, company size, and nature, the analysis designated work gamification as the independent variable and creativity as the dependent variable. In Model 2, the findings show that work gamification positively influences employee creativity (β = 0.78, *p* < 0.001), affirming Hypothesis 1. In Model 4, including both work gamification and intrinsic motivation as independent variables, with creativity as the dependent variable, the analysis found intrinsic motivation also positively affects employee creativity (β = 0.286, *p* < 0.001), thereby supporting Hypothesis 3. Taking work gamification as the independent variable and intrinsic motivation as the dependent variable, the results in Model 6 demonstrate a positive influence on intrinsic motivation (β = 0.693, *p* < 0.001), thereby supporting Hypothesis 2.

**Table 6:** Regression analysis outcomes.

**Variable**	**Creativity**				**Intrinsic motivation**	
	**Mode l**	**Mode 2**	**Mode 3**	**Mode 4**	**Mode 5**	**Mode 6**
**Control variable**						
Gender	0.201	−0.056	0.314	0.070	0.203	0.075
Age	0.237^**^	0.092	0.105	−0.008	−0.017	0.211^***^
Education	−0.205	0.052	−0.194	−0.003	0.037	−0.014
Company size	0.051	−0.007	0.027	−0.026	0.023	−0.014
Company nature	0.012	−0.030	−0.003	−0.028	−0.097	−0.011
**Independent variable**						
Work gamification		0.780^***^		0.582^***^		0.693^***^
**Mediator variable**						
Intrinsic motivation			0.647^***^	0.286^***^		
*R* ^2^	0.067	0.601	0.477	0.646	0.022	0.443
Δ*R*^2^	0.044	0.589	0.462	0.634	−0.001	0.427
*F*	3.007^**^	52.642^***^	31.873^***^	54.536^***^	0.955	27.791^***^

To further ascertain the mediating effect of intrinsic motivation, the study employed a bootstrap test with 5,000 resamples via SPSS. Incorporating these variables into the model, the confidence interval for the mediating effect of intrinsic motivation on creativity, induced by work gamification, was (0.6884, 0.8719), excluding 0 (as shown in Table 7). This finding supports Hypothesis 4, indicating a significant mediating role of intrinsic motivation in the relationship between work gamification and employee creativity.

**Table 7:** Mediation effect analysis of intrinsic motivation.

	**Effect**	**se**	***t***	***p***	**LLCI**	**ULCI**
Total effect	0.7801	0.0465	16.7593	0.0000	0.6884	0.8719
Direct effect	0.5822	0.0582	10.0092	0.0000	0.4675	0.6969
Indirect effect	0.1979	0.0607			0.0879	0.3264

The above analysis demonstrates that work gamification exerts positive effects on employees' intrinsic motivation and creativity. Furthermore, intrinsic motivation positively influences employee creativity and serves as a mediating factor in the relationship between work gamification and employee creativity.

#### 4.5.2 Indirect effect analysis

**Figure 3:**
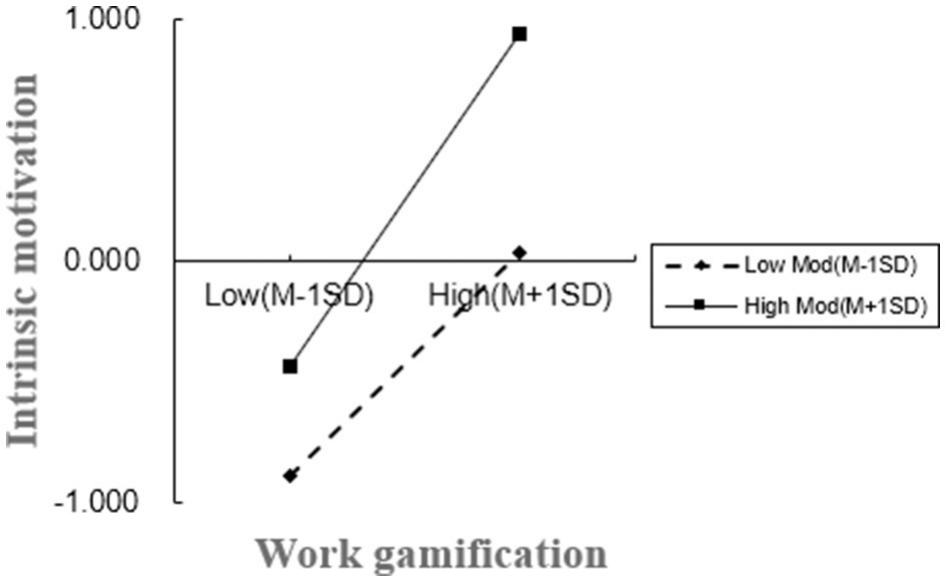
The moderating effect of the level of acceptance of work gamification on the relationship between work gamification and intrinsic motivation.

Significant interaction effects were found between work gamification and its acceptance levels on intrinsic motivation (β = 0.1126, *p* < 0.01). At higher acceptance levels, work gamification markedly enhances intrinsic motivation; conversely, at lower levels, its positive effect, while significant, is comparatively weaker, as shown in Figure 3. This supports Hypothesis 5.

To substantiate Hypothesis 6, a moderated mediation analysis utilizing the Bootstrap method was performed (as shown in Table 8). The analysis revealed that at low acceptance levels, work gamification significantly mediates creativity through intrinsic motivation [95%CI = (0.0524, 0.2429)], yielding a mediation effect of 0.1319. Similarly, at high acceptance levels, the mediation of creativity by intrinsic motivation remains significant [95%CI = (0.0901, 0.3183)], with an effect value of 0.1962. Significantly, the difference in mediation effects between high and low acceptance levels is confirmed [95%CI = (0.0001, 0.0666)], with an effect difference of 0.0322, thereby supporting Hypothesis 6.

**Table 8:** Results of moderated mediation analysis.

**The level of acceptance of work gamification**	**Effect**	**BootSE**	**BootLLCI**	**BootULCI**
Interaction term	0.1126	0.0402	0.0334	0.1919
−1	0.1319	0.0489	0.0524	0.2429
0	0.164	0.0507	0.0745	0.2728
1	0.1962	0.0578	0.0901	0.3183
Moderated mediation	0.0322	0.0171	0.0001	0.0666

The above analysis indicates that the level of acceptance of work gamification moderates the relationship between work gamification and intrinsic motivation. Furthermore, it positively moderates the mediating role of intrinsic motivation in the link between work gamification and employee creativity.

## 5 Discussion

### 5.1 Key findings

This study, grounded in flow theory, investigated how work gamification influences employee creativity through intrinsic motivation and examined the moderating role of the level of acceptance of work gamification. Empirical results demonstrate that: Work gamification exerts a significant positive impact on employee creativity (H1 supported); Intrinsic motivation fully mediates this effect (H2–H4 supported: β = 0.42, *p* < 0.001; β = 0.36, *p* < 0.001; β = 0.31, *p* < 0.001); The level of acceptance of work gamification significantly moderates the relationship between gamification and intrinsic motivation (H5 supported: interaction term β = 0.27, *p* < 0.01); Acceptance level further moderates the mediating effect of intrinsic motivation (H6 supported: moderated mediation index = 0.14, *p* < 0.05). Collectively, these findings confirm the critical role of work gamification in enhancing creativity while highlighting intrinsic motivation and employee acceptance as pivotal influencing factors.

### 5.2 Theoretical implications

First, it enriches the theoretical framework of how work gamification drives employee creativity from a flow theory perspective. By creating environments with clear goals, immediate feedback, and balanced challenges, work gamification triggers flow experiences that enhance intrinsic motivation and creativity—aligning with Lieberoth (2015), Banfield and Wilkerson (2014), and Bakker and Demerouti (2007). Crucially, it establishes intrinsic motivation as the core mediating mechanism.

Second, the introduction and validation of the level of acceptance of work gamification addresses a critical research gap regarding individual differences. Results confirm that higher acceptance amplifies the positive effects on intrinsic motivation and creativity, resonating with Mollick and Rothbard (2014) assertion that employee buy-in determines gamification effectiveness.

Third, conducting this research in China extends the cross-cultural validation of flow theory and self-determination theory, demonstrating their applicability within Confucian cultural contexts while delineating boundary conditions (Huang and Bond, 2012).

### 5.3 Management insights

First, strengthen intrinsic motivation to unlock employees' creative potential. Enterprises should prioritize employees' intrinsic motivation by designing gamified tasks with clear goals, timely feedback, and appropriate challenges. This fulfills psychological needs for autonomy, competence, and relatedness, thereby enabling sustained enhancement of creativity.

Second, implement differentiated management to elevate employees' acceptance of work gamification. During recruitment and training, enterprises should assess employees' receptiveness to new approaches. High-acceptance employees can be identified through evaluations and leveraged as change agents to facilitate the integration of lower-acceptance employees into the new work paradigm.

Third, continuously optimize design by emphasizing intrinsic reward mechanisms. Enterprises must transcend extrinsic incentive models (points, badges, leaderboards) and increasingly incorporate gamified narratives and contextual experiences. This creates intrinsically engaging work environments that stimulate persistent interest and commitment (Hamari et al., 2014).

Finally, foster supportive climates to cultivate fertile ground for innovation. Leaders should cultivate work environments emphasizing autonomy, trust, and support. This encourages proactive exploration and bold innovation, while providing sufficient resources and safeguards to ensure the smooth implementation of creative initiatives.

It is important to note that the effectiveness of work gamification may vary across industries. In technology companies, gamification often integrates digital platforms and innovation-driven approaches, emphasizing immediate feedback and personalized challenges, making it suitable for highly autonomous and knowledge-intensive work environments. In manufacturing, however, gamification primarily focuses on motivating standardized processes and enhancing team collaboration, where operational safety and efficiency must be balanced. Designs in this sector should prioritize simplicity, ease of use, and on-site interactive experiences. Enterprises should flexibly adapt gamification strategies based on industry characteristics to maximize motivational impact and creativity enhancement.

Furthermore, it is crucial to note that excessive gamification may introduce negative consequences. Overreliance on extrinsic incentives could undermine employees' intrinsic motivation, while overly competitive elements may increase stress levels or even lead to burnout. Therefore, when implementing gamification, organizations should strike a reasonable balance between intrinsic and extrinsic motivators, prioritize employee psychological wellbeing, and avoid the detrimental effects of “forced gamification.”

### 5.4 Shortcomings and prospects

First, the reliance on online surveys for data collection may impose limitations regarding sample representativeness and data authenticity. Future research should integrate interviews, experimental designs, and objective behavioral data to enhance the robustness of findings.

Second, the use of cross-sectional data hinders the examination of dynamic changes in work gamification effects. Subsequent studies should employ longitudinal tracking or time-series analysis to holistically investigate the long-term impact of gamification designs on creativity over time.

Third, given the study's grounding in China's unique cultural context, cultural differences may influence the acceptance and effectiveness of gamification designs. Future cross-cultural research should compare attitudes and responses toward work gamification across countries/regions to further explore cultural boundaries of gamification applications.

Finally, individual employee traits and task-contextual factors may serve as critical moderators. Future research should incorporate individual variables (e.g., performance level, age, self-efficacy, competitiveness) and contextual variables (e.g., task complexity, skill-task match) to further clarify the applicability boundaries of work gamification.

In conclusion, this study achieved its research objectives by empirically validating work gamification's positive influence on creativity, establishing intrinsic motivation as the core mediator, and identifying employee acceptance as a critical boundary condition—advancing both theoretical and practical understanding.

## Data Availability

The raw data supporting the conclusions of this article will be made available by the authors, without undue reservation.

## References

[B1] Amabile T. M. Barsade S. G. Staw M. B. M. (2005). Affect and creativity at work. Soc. Sci. Electron. Publ. 50, 367–403. 10.2189/asqu.2005.50.3.36721821037

[B2] Amabile T. M. Hill K. G. Hennessey B. A. Tighe E. M. (1994). The work preference inventory: assessing intrinsic and extrinsic motivational orientations. J. Pers. Soc. Psychol. 66, 950–967. 10.1037/0022-3514.66.5.950 8014837

[B3] Bakker A. B. Demerouti E. (2007). The job demands-resources model: state of the art. J Manag. Psychol. 22, 309–328. 10.1108/0268394071073311531861812

[B4] Banfield J. Wilkerson B. (2014). Increasing student intrinsic motivation and self-efficacy through gamification pedagogy. Contemp. Iss. Educ. Res. 7:291. 10.19030/cier.v7i4.8843

[B5] Behl A. Jayawardena N. Ishizaka A. Gupta M. Shankar A. (2022). Gamification and gigification: a multidimensional theoretical approach. J. Bus. Res. 139, 1378–1393. 10.1016/j.jbusres.2021.09.023

[B6] Bentler P. M. Chou C. P. (1987). Practical issues in structural modeling. Sociol. Methods Res. 16, 78–117. 10.1177/0049124187016001004

[B7] Bitrián P. Buil I. Catalán S. Hatfield S. (2023). The use of gamification strategies to enhance employees' attitudes towards e-training systems. Int. J. Manag. Educ. 17:100892. 10.1016/j.ijme.2023.100892

[B8] Brislin R. W. (1986). The Wording and Translation of Research Instruments. Los Angeles, CA: Sage.

[B9] Cardador M. T. Northcraft G. B. Whicker J. (2017). A theory of work gamification: something old, something new, something borrowed, something cool? Hum. Resour. Manag. Rev. 10.1016/j.hrmr.2016.09.014

[B10] Chen H. Wigand R. T. Nilan M. (2000). Exploring web users' optimal flow experiences. Inf. Technol. People 13, 263–281. 10.1108/0959384001035947324776786

[B11] Chen Y. Gao L. (2021). Can gamification motivate employees' proactive behavior? Foreign Econ. Manag. 43, 133–152. 10.16538/j.cnki.fem.20210617.301

[B12] Cohen J. (1992). A power primer. Psychol. Bull. 112, 155–159. 10.1037/0033-2909.112.1.155 19565683

[B13] Comrey A. L. Lee H. B. (1992). ^*^A First Course in Factor Analysis^*^, 2nd Edn. Hillsdale, NJ: Lawrence Erlbaum Associates.

[B14] Csikszentmihalyi M. (1991). Flow: The Psychology of Optimal Experience. New York, NY: HarperPerennial.

[B15] Danis W. Dollinger M. (1998). A provisional comparison of factor structures using english, japanese, and chinese versions of. Psychol. Rep. 83:1095. 10.2466/pr0.1998.83.3.1095

[B16] Deci E. L. Ryan R. M. (2013). Intrinsic Motivation and Self-Determination in Human Behavior. New York, NY: Springer Science and Business Media.

[B17] Deterding S. Dixon D. Khaled R. Nacke L. (2011). “From game design elements to gamefulness: defining “gamification”,” in Proceedings of the 15th International Academic MindTrek Conference: Envisioning Future Media Environments (New York, NY), 9–15.

[B18] Dichev C. Dicheva D. (2017). Gamifying education: what is known, what is believed and what remains uncertain: a critical review. Int. J. Educ. Technol. High. Educ. 14, 1–36. 10.1186/s41239-017-0042-5

[B19] Florin O. Christian J. Mary K. (2014). I play at work: ten principles for transforming work processes through gamification. Front. Psychol. 5:14. 10.3389/fpsyg.2014.00014 24523704 PMC3906598

[B20] Friedrich J. Becker M. Kramer F. Wirth M. Schneider M. (2019). Incentive design and gamification for knowledge management. J. Bus. Res. 106, 341–352. 10.1016/j.jbusres.2019.02.009

[B21] Fuller J. B. Hester M. K. (2010). Promoting felt responsibility for constructive change and proactive behavior: exploring aspects of an elaborated model of work design. J. Organ. Behav. 27, 1089–1120. 10.1002/job.408

[B22] Glynn A. M. (1994). Effects of work task cues and play task cues on information processing, judgment, and motivation. J. Appl. Psychol. 79, 34–45. 10.1037/0021-9010.79.1.34 8200873

[B23] Gulzar M. A. Ahmad M. Hassan M. Rasheed M. I. (2021). How social media use is related to student engagement and creativity: investigating through the lens of intrinsic motivation. Behav. Inf. Technol. 40, 1–11. 10.1080/0144929X.2021.1917660

[B24] Gursesli M. C. Martucci A. Mattiassi A. D. Duradoni M. Guazzini A. (2024). Development and validation of the psychological motivations for playing video games scale (PMPVGs). Simul. Gaming 55, 856–885. 10.1177/10468781241260861

[B25] Hagtvedt L. P. Dossinger K. Harrison S. H. Huang L. (2019). Curiosity made the cat more creative: specific curiosity as a driver of creativity. Organ. Behav. Hum. Decis. Process. 150, 1–13. 10.1016/j.obhdp.2018.10.007

[B26] Hamari J. Koivisto J. (2014). Measuring flow in gamification: dispositional flow scale-2. Comput. Human Behav. 40, 133–143. 10.1016/j.chb.2014.07.048

[B27] Hamari J. Koivisto J. Sarsa H. (2014). “Does gamification work? — A literature review of empirical studies on gamification,” in The 47th Hawaii International Conference on System Sciences (Piscataway, NJ: IEEE).

[B28] Han W. Feng X. Zhang M. Peng K. Zhang D. (2019). Mood states and everyday creativity: employing an experience sampling method and a day reconstruction method. Front. Psychol. 10:1698. 10.3389/fpsyg.2019.01698 31379699 PMC6658875

[B29] Huang X. Bond M. H. (eds.). (2012). Handbook of Chinese Organizational Behavior: Integrating Theory, Research and Practice. Cheltenham: Edward Elgar Publishing.

[B30] Hung C. Y. Sun C. Y. Yu P. T. (2015). The benefits of a challenge: student motivation and flow experience in tablet-pc-game-based learning. Interact. Learn. Environ. 23, 172–190. 10.1080/10494820.2014.99724827885969

[B31] Inal Y. Cagiltay K. (2007). Flow experiences of children in an interactive social game environment. Br. J. Educ. Technol. 38, 455–464. 10.1111/j.1467-8535.2007.00709.x

[B32] Jiang T. Chen P. Xu Y. (2021). Progress in the application research of flow theory abroad. J. Inf. Resour. Manag. 11, 4–16. 10.13365/j.jirm.2021.01.00417209405

[B33] Jie M. Wenyuan W. (2023). Curiosity causes creativity? Revealing the reinforcement circle between state curiosity and creativity. J. Creat. Behav. 54, 940–947. 10.1002/jocb.606

[B34] Johnson D. Deterding S. Kuhn K. A. Staneva A. Stoyanov S. Hides L. (2016). Gamification for health and wellbeing: a systematic review of the literature. Int. Intervent. 6, 89–106. 10.1016/j.invent.2016.10.002 30135818 PMC6096297

[B35] Kang Y. Zheng Q. (2023). Study on the impact of work curiosity and creative process engagement on job performance: a theoretical analysis framework. Modern Manag. 13, 1782–1791. 10.12677/MM.2023.1312224

[B36] Keller J. Landhaeusser A. (2011). Experiencing flow: experimental analyses of the experiential state resulting from optimal task demands. Psychologische Rundschau 62:58. 10.1026/0033-3042/a000058

[B37] Kiili K. (2005). Digital game-based learning: towards an experiential gaming model. Int. High. Educ. 8, 13–24. 10.1016/j.iheduc.2004.12.001

[B38] Koivisto J. Hamari J. (2019). The rise of motivational information systems: a review of gamification research. Int. J. Inf. Manag. 45, 191–210. 10.1016/j.ijinfomgt.2018.10.01340062254

[B39] Lieberoth A. (2015). Shallow gamification testing psychological effects of framing an activity as a game. Games Culture 10, 229–248. 10.1177/1555412014559978

[B40] Liu J. (2017). Modeling and Verification of the Gamification in Education (Doctoral dissertation). East China Normal University. Available online at: https://kns.cnki.net/kcms2/article/abstract?v=a4fp6zKrpgbucUY1ve4zrqPFFt_cExMK-pEeVvc2hLC6ukY5DdmivztBrvRqyopY8mKYu9SXEpBtKuAFrDMcLnHPQQveNifWAMWY8M9JVk7K7ZNoYA4zg0QGAsehRoCBNLOVn0dCLYr_qp7qnfUp1rzz70QbXu5F-k1eZiHsOPerpgOP1jzyTKP7w3QPuMm-8wgXMJ8z9z4=&uniplatform=NZKPT&language=CHS (Accessed June 23, 2025).

[B41] LoVoll H. S. Vitters J. (2014). Can balance be boring? A critique of the “challenges should match skills” hypotheses in flow theory. Soc. Indic. Res. 115, 117–136. 10.1007/s11205-012-0211-9

[B42] Marylène G. Deci E. L. (2005). Self-determination theory and work motivation. J. Organ. Behav. 26, 331–362. 10.1002/job.322

[B43] Meng L. (2016). Task Design Incorporating Self-Determination Theory and One's Intrinsic Motivation: An Empirical Investigation Form a Cognitive Neuroscience Perspective (Doctoral dissertation). Zhejiang University. Available online at: https://kns.cnki.net/kcms2/article/abstract?v=Z-eERPAUDzySfv15w-ugqcawqyeZtOPNwTYidMdZXT7PAjfImOVjh_yazHtLNBytJa2R2qhg-JvJ5vvRGQCPwPUCQdopwr8hmStqacoNbkQLSZMWP2oS0DEwOHYSZJb4-Brn2j0dnw-sj2slT_RAxjSaWZuTxu_Pv0FAiqJ_oZ81PkoTv-D1lfeKrM05AZbzeJP7reu-eNc=anduniplatform=NZKPTandlanguage=CHS (Accessed June 23, 2025).

[B44] Mitchell R. Schuster L. Jin H. S. (2020). Gamification and the impact of extrinsic motivation on needs satisfaction: making work fun? J. Bus. Res. 106, 465–476. 10.1016/j.jbusres.2018.11.022

[B45] Mollick E. R. Rothbard N. (2013). Mandatory Fun: Consent, Gamification and the Impact of Games at Work. Rochester, NY: Social Science Electronic Publishing.

[B46] Mollick E. R. Rothbard N. (2014). Mandatory Fun: Consent, Gamification and the Impact of Games at Work. The Wharton School Research Paper Series.

[B47] Novak T. P. Hoffman D. L. Yung Y. F. (2000). Measuring the customer experience in online environments: a structural modeling approach. Market. Sci. 19, 22–42. 10.1287/mksc.19.1.22.1518419642375

[B48] Novak T. P. Hoffman D. L. Duhachek A. (2003). The influence of goal-directed and experiential activities on online flow experiences. J. Consum. Psychol. 13, 3–16. 10.1207/153276603768344744

[B49] Palomki J. Tammi T. Lehtonen N. Seittenranta N. Cowley B. U. (2021). The link between flow and performance is moderated by task experience. Comput. Human Behav. 124:106891. 10.1016/j.chb.2021.106891

[B50] Patrício R. Moreira A. Francesco Z. (2018). Gamification approaches to the early stage of innovation. Creat. Innovat. Manag. 27:12284. 10.1111/caim.12284

[B51] Robson K. Plangger K. Kietzmann J. H. Mccarthy I. Pitt L. (2016). Game on: engaging customers and employees through gamification. Bus. Horiz. 59, 29–36. 10.1016/j.bushor.2015.08.002

[B52] Ryan R. M. Deci E. L. (2000). Self-determination theory and the facilitation of intrinsic motivation, social development, and well-being. Am. Psychol. 55, 68–78. 10.1037/0003-066X.55.1.68 11392867

[B53] Sailer M. Homner L. (2020). The gamification of learning: a meta-analysis. Educ. Psychol. Rev. 32, 77–112. 10.1007/s10648-019-09498-w

[B54] Sardi L. Idri A. Fernández-Alemán J. L. (2017). A systematic review of gamification in e-health. J. Biomed. Inform. 71, 31–48. 10.1016/j.jbi.2017.05.011 28536062

[B55] Schutte N. S. Malouff J. M. (2020). A Meta-Analysis of the Relationship Between Curiosity and Creativity. Hoboken, NJ: John Wiley and Sons, Ltd.

[B56] Shanshan Q. (2025). Optimizing the learning experience guided by flow theory: a case study of practical exercise in tourism higher education.journal of hospitality and tourism. Education 37, 126–137. 10.1080/10963758.2025.2456636

[B57] Silvia P. J. Winterstein B. P. Willse J. T. Barona C. M. Cram J. T. Hess K. I.. (2008). Assessing creativity with divergent thinking tasks: exploring the reliability and validity of new subjective scoring methods. meeting of the midwestern psychological association, 2007, this research was presented at the aforementioned conference. Psychol. Aesthet. Creat. Arts 2, 68–85. 10.1037/1931-3896.2.2.68

[B58] Smith R. Popa D. (2015). Why play matters at work: gamification is more than just a passing fad. IEEE Cons. Electron. Mag. 4, 73–79. 10.1109/MCE.2015.2421574

[B59] Tian Y. Ou L. J. (2023). How do personality traits of college students affect their learning flow experience? Learn. Motiv. 10.1016/j.lmot.2023.101917

[B60] Tierney P. Farmer S. M. Graen G. B. (1999). An examination of leadership and employee creativity: the relevance of traits and relationships. Pers. Psychol. 52, 591–620. 10.1111/j.1744-6570.1999.tb00173.x20417972

[B61] Wangsupa L. C. Chensupa M. P. (2010). The effects of game strategy and preference-matching on flow experience and programming performance in game-based learning. Innovat. Educ. Teach. Int. 47, 39–52. 10.1080/14703290903525838

[B62] Xi N. Hamari J. (2019). Does gamification satisfy needs? A study on the relationship between gamification features and intrinsic need satisfaction. Int. J. Inf. Manag. 46, 210–221. 10.1016/j.ijinfomgt.2018.12.00234651180

[B63] Yidong T. Xinxin L. (2013). How ethical leadership influence employees' innovative work behavior: a perspective of intrinsic motivation. J. Bus. Ethics 116, 441–455. 10.1007/s10551-012-1455-7

[B64] Zikos S. Tsourma M. Lithoxoidou E. E. Drosou A. Ioannidis D. Tzovaras D. (2019). User acceptance evaluation of a gamified knowledge sharing platform for use in industrial environments. Int. J. Serious Games 6, 89–108. 10.17083/ijsg.v6i2.275

